# 

**Published:** 1877-03

**Authors:** 


					TO READERS AND (JORKKSI'ON DEN'liS.
Communications intended for publication, and exchange journals and pape
should be addressed to the Editor of the American Journal of Dent.
Science 259 N. Eutaw ?t., Baltimore, Md. All letters relating to the busine
of the journal, or containing cash remittances, to be directedto Messrs. Snowd:
& Cowman. Articles for publication should be received by the editor at le
three weeks previous to the first day of the month on which the number in whi
it, is designed for them to appear, is issued.
1
f^r?The American Journal of Dental Science will contain fifty pages o* I
reading matter in each number, making a volume of not less than
Six Hundred pages
EXCLUSIVE OF ADVERTISEMENTS
T 11 t<.
\ME1UCAN JOURNAL
OF
DENTAL SCIENCE.
F. J. S. GORGAS, M.D.,D.D.S., Editor.
The Journal is issued on the first of each month and contains not less
than fifty pages of reading matter in each number, exclusive of adver-
tisements, making a volume of six hundred pages per annum.
In each number will be found Original Communications, Corres-
pondence, Selected Articles, Monthly Summary, Bibliographical No-
tices and Editorial Matter, and every effort will be exerted to render
the Journal worthy of the patronage of the Dental profession.
A generous co-operation is respectfully solicited from the members
of the profession ; and as the Journal has now a printing omce of its
own, which materially lessens the cost 01 its publication, it will be
issued at the reduced subscription price of
I.SO Per Annum in Advance.
Communications are respectfully solicited on all subjects pertain-
ing to the practice of dentistry, as well as reports oi cases occurring
in dental practice.
RATES OF ADVERTISING.
1 Page.
* "
'A"
1 MO.
$15 00
10 00
7 00
$ 00
2 MO.
$22 50
15 00
11 00
8 00
3 MO.
$30 00
20 00
15 00
11 00
6 MO.
$50 00
30 00
20 00
15 00
12 MO.
$100 00
50 00
30 00
20 00
All original communications designed for publication, works for
review, new instruments for notice, exchanges, &c., should be directed
to the editor, 259 N. Eutaw St., Baltimore, Md.
All advertisements and subscriptions should be addressed to
SNOWDEN & COWMAN,
Publishers of the Am. Journal of Dental Science.
86 W. Fayette St. Bet. Charles & Liberty Sts.,
BALTIMORE, MD.

				

